# Comparative Transcriptome Analysis in Eggplant Reveals Selection Trends during Eggplant Domestication

**DOI:** 10.1155/2019/7924383

**Published:** 2019-05-09

**Authors:** Qingzhen Wei, Liming Du, Wuhong Wang, Tianhua Hu, Haijiao Hu, Jinglei Wang, Karine David, Chonglai Bao

**Affiliations:** ^1^Institute of Vegetable Research, Zhejiang Academy of Agricultural Sciences, Hangzhou 30021, China; ^2^The University of Auckland, School of Biological Sciences, Private Bag 91019, Auckland 1010, New Zealand

## Abstract

Eggplant (*Solanum melongena* L.) is an economically and nutritionally important fruit crop of the Solanaceae family, which was domesticated in India and southern China. However, the genome regions subjected to selective sweeps in eggplant remain unknown. In the present study, we performed comparative transcriptome analysis of cultivated and wild eggplant species with emphasis on the selection pattern during domestication. In total, 44,073 (*S. sisymbriifolium*) to 58,677 (*S. melongena* cultivar S58) unigenes were generated for the six eggplant accessions with total lengths of 36.6-46 Mb. The orthologous genes were assessed using the ratio of nonsynonymous (*K*_a_) to synonymous (*K*_s_) nucleotide substitutions to characterize selective patterns during eggplant domestication. We identified 19 genes under positive selection across the phylogeny that were classified into four groups. The gene (*OG12205*) under positive selection was possibly associated with fruit-related traits in eggplant, which may have resulted from human manipulation. Eight positive selected genes were potentially involved in stress tolerance or disease resistance, suggesting that environmental changes and biotic stresses were important selective pressures in eggplant domestication. Taken together, our results shed light on the effects of artificial and natural selection on the transcriptomes of eggplant and its wild relatives. Identification of the selected genes will facilitate the understanding of genetic architecture of domesticated-related traits and provide resources for resistant breeding in eggplant.

## 1. Introduction

Over human history, wild plants and animals were selected and adapted for cultivation and commercial use, a process known as domestication. Darwin proposed that domestication and adaptation were in parallel in the wild and are powerful in generating phenotypic diversity [1]. Selection in the wild and during domestication follows similar processes, which involves the selection of beneficial alleles present in the wild germplasm or arose *via* spontaneous mutations at a collection of loci controlling yield and quality. Studies on several organisms suggest that very few genetic loci contributed to the rapid phenotypic divergence relative to domestication [2–6].

With the improvements of sequencing technologies and increasing availability of plant genomes, the consequences of domestication could be studied at a transcriptional or even whole-genome level. Several domestication-associated genes were characterized in some crops such as rice, maize, tomato, and pepper [7–10]. A recent study in maize showed that 46 genes were putative targets of selection with functions mostly related to biotic stress [9]. In tomato, around 50 genes were under positive selection during domestication, the adaptation to extreme environments caused a broad alteration of transcriptional networks, and the sequences of genes were involved in environmental and stress responses [8]. Despite the importance of the absolute changes in gene expression or changes in regulation networks during domestication, the selective patterns among domesticated species and multiple wild relatives are still limited to a few organisms.

Brinjal eggplant (*Solanum melongena* L., 2*n* = 2*x* = 24) is an economically important vegetable crop and is widely grown in America, Europe, and Asia, with 51.3 million tons of agricultural production in 2016 (http://faostat.fao.org). The eggplant belongs to the large family Solanaceae, which compromises >3000 species with diverse genetic and phenotypic variation including tomato, pepper, and potato. Eggplants exhibit a wide biodiversity of local landraces/wild relatives with huge variation respect to growth habit, pathogen resistance, and fruit size and color. The subgenus *Leptostemonum* (“spiny solanums”) comprises approximately 500 species, including three closely related cultivated eggplant species: *S. melongena* (brinjal eggplant, section Melongena), *S. macrocarpon* (gboma eggplant, section Melongena), and *S. aethiopicum* (scarlet eggplant, section Oliganthes), which are all of Old World origin and grown as food crops [11]. The domestication region of eggplant has long been debated; two most commonly hypothesized regions are India and southern China/Southeast (SE) Asia [12], where eggplant use has equally old written records dating approximately 2000 years ago. Both of the hypothesized regions have highly diverse landraces and populations of candidate progenitors of domesticated eggplants (*S. incanum* L. in India and *S. undatum* L. in southern China). Domesticated eggplant exhibits substantial phenotypic differences from its wild progenitors, reflecting rapid and pronounced evolutionary changes. Identification of genetic changes underlying these phenotypic differences will give insight into the genetic architecture of complex traits, the response to selection, and provide resources for eggplant improvement.

The availability of the eggplant genome (*S. melongena* cv. Nakate-Shinkuro) [13] provides an excellent opportunity for performing comparative analysis at the transcriptome level. In recent years, various studies have been performed on comparative transcriptomics to investigate molecular mechanisms underlying disease-resistant, fruit-related traits and develop molecular markers in eggplant [14–17]. However, the knowledge of changes in gene expression or transcriptional regulation networks during domestication is still lacking. In the present study, we investigated gene divergence accompanied with eggplant domestication by comparative transcriptomics between wild and domesticated eggplants. We examined the transcriptomes of three cultivated eggplant accessions (S58, HZHQ, and LYQ) and three wild relatives (*S. aethiopicum*, *S. integrifolium*, and *S. sisymbriifolium*). Evidence for significant changes in both gene expression levels and sequences was found which might have occurred as a result of selective sweeps and specifically identified a subset of genes that were likely targets of selection during domestication.

## 2. Materials and Methods

### 2.1. Plant Materials, Growth, and RNA Isolation

**Figure 1:**
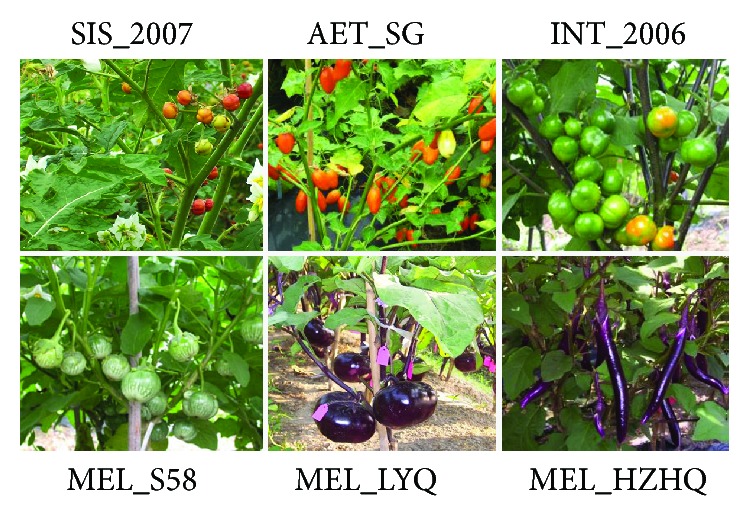
The fruit and leaf morphology of eggplant (*Solanum melongena*) and wild relatives. AET_SG: *S. aethiopicum* L.; SIS_2007: *S. sisymbriifolium* L.; INT_2006: *S. integrifolium*; MEL_S58, MEL_HZHQ; MEL_LYQ: *S. melongena*.

Six different eggplants were used in the present study, three wild species (*S. sisymbriifolium*, *S. aethiopicum*, and *S. integrifolium*) and three domesticated eggplant cultivars from *S. melongena* (S58, HZHQ, and LYQ) (Figure 1). Two eggplant cultivars, HZHQ and LYQ, are widely grown across China (HZHQ in Zhejiang and LYQ in Beijing); both are purple black-skinned and have either a long fruit or an oval fruit (Figure 1). The seeds of HZHQ and LYQ were collected by the Institute of Vegetable Sciences (Zhejiang Academy of Agricultural Sciences), whereas the seeds of eggplant cultivar S58 were provided by the Asian Vegetable Research and Development Center (http://www.avrdc.org, Thailand branch), and it is cultivated in Thailand. The seeds of *S. sisymbriifolium*, *S. aethiopicum*, and *S. integrifolium* were obtained from Xishuangbanna Botanical Garden (Yunnan, China). *S. aethiopicum* is mostly grown in tropical Africa [18]; however, *S. aethiopicum* has four classifications (Gilo, Shum, Kumba, and Aculeatum); *S. integrifolium* (*S. aethiopicum* gr. *Aculeatum*) is one of them and mainly used as rootstocks (mansfeld.ipk-gatersleben.de). *S. aethiopicum* and *S. integrifolium* belong to the section Oliganthes which forms a sister group to *S. melongena* within the Old World clade [12], whereas *S. sisymbriifolium* belongs to the Sisymbriifolium clade, distant from the Old World clade [19].

Seeds (10 per species) from the six eggplants were germinated in soil and in the dark for 7 days. Plants were then transferred into growth chambers (28°C/22°C day/night temperatures and 14 h photoperiod with 300 *μ*mol photons m^−2^ s^−1^). When the plantlets had three true leaves, the leaf tissue was collected from the seedlings 14 days postgermination. The leaf tissue for each species was collected from three individual plants then mixed for RNA extraction. Total RNA of each sample was extracted using an RNAprep Pure Plant Kit (DP432, TIANGEN, China, http://www.tiangene.com) according to the manufacturer's protocol.

### 2.2. cDNA Library Construction, Sequencing, and Assembly

Illumina sequencing was performed at Novogene Bioinformatics Technology Co. Ltd., Beijing, China (http://www.novogene.cn). mRNA was purified from 3 *μ*g total RNA using poly-T oligo-attached magnetic beads. Sequencing libraries were generated using Illumina TruSeq™ RNA Sample Preparation Kit (Illumina, San Diego, USA) following the manufacturer's recommendations. In order to select cDNA fragments of preferentially 200 bp in length, the library fragments were purified with the AMPure XP system (Beckman Coulter, Beverly, USA), then sequenced on an Illumina Hiseq 2000 platform, and 90 bp paired-end reads were generated. Clean reads were obtained by removing reads containing adapter and poly-N and low-quality reads from raw data. All the downstream analyses were based on clean data with high quality. Transcriptome assembly was accomplished based on the left files and right files using Trinity [20] with min_kmer_cov set to 2 by default and all other parameters set by default.

### 2.3. Annotation, GO, and KEGG Pathway Enrichment Analysis

To annotate the unigenes of the eggplant transcriptomes, each of the unigenes from all six eggplants was searched against different public databases, including KEGG Orthology database, Gene Ontology (GO) database, NCBI nonredundant protein sequences (NR) database, protein family (PFAM) database, Swiss-Prot protein database, NCBI nucleotide sequence (NT) database, and Eukaryotic Orthologous Group (KOG) database. GO enrichment analysis of genes was implemented by the GOseq R package-based Wallenius noncentral hypergeometric distribution [21], which can adjust for gene length bias. KEGG [22] is a database resource for understanding high-level functions and utilities of the biological system, such as cell, organism, and ecosystem, from molecular-level information, especially large-scale molecular datasets generated by genome sequencing and other high-throughput experimental technologies (http://www.genome.jp/kegg/). We used KOBAS [23] software to test the statistical enrichment of differential expression genes in KEGG pathways.

### 2.4. Alignment of RNA-seq Data to the Reference Eggplant Genome

Reference genome and gene model annotation files were directly downloaded from the eggplant genome database (http://eggplant.kazusa.or.jp/). The index of the reference genome was built using Bowtie v2.0.6, and paired-end clean reads were aligned to the reference genome using TopHat v2.0.9. We selected TopHat as the mapping tool for TopHat can generate a database of splice junctions based on the gene model annotation file and thus a better mapping result than other nonsplice mapping tools.

The Cufflinks v2.1.1 Reference Annotation-Based Transcript (RABT) assembly method was used to construct and identify both known and novel transcripts from TopHat alignment results. Picard-tools v1.96 and SAMtools v0.1.18 were used to sort, mark duplicated reads, and reorder the bam alignment results of each sample.

### 2.5. Quantification of Gene Expression Levels

Gene expression levels were estimated by RSEM [24] for each sample. Clean data were mapped back onto the assembled transcriptome, and HTSeq v0.5.3 (EMBL, Heidelberg, Germany) was used to count the read numbers mapped to each gene. RPKM (reads per kilobase of exon model per million mapped reads) considers the effect of sequencing depth and gene length for the read count at the same time and is currently the most commonly used method for estimating gene expression level [25].

### 2.6. Validation of RNA-seq Data by Real-Time Quantitative PCR

RNA-seq data for 16 different genes was validated by real-time quantitative PCR (qPCR). The primers of selected genes were designed using Primer Priemer 5 software (PREMIER Biosoft, Palo Alto, CA, USA) and synthesized by Sangon. The primer pair SmActin-F and SmActin-R was used to amplify an eggplant actin fragment as a control for normalizing the starting amounts of cDNA. The sequences of primers used in this study are listed in Supplementary Table S4. The first-strand cDNA was synthesized using 1 *μ*g total RNA by HiScript II Q Select RT SuperMix for qPCR (+gDNA wiper) (Vazyme, Nanjing, China) in 10 *μ*L of reaction mixture. The quantitative PCRs were performed according to the manufacturer's instructions using the DyNAmo™ Flash SYBR Green qPCR Kit (Tiangen Biotech, Beijing, China) in an ABI Prism StepOnePlus real-time thermal cycler (Applied Biosystems, Carlsbad, CA, USA). The amplification was performed as follows: 10 min at 94°C followed by 40 cycles of 94°C for 20 s and 60°C for 60 s. A melting curve was generated to ensure product uniformity. Gene expression was evaluated by the 2^_△△^Ct method [26]. The expression of genes was related to the SmActin expression. The correlation between expression profiles of selected genes obtained from real-time PCR and RNA-seq data based on log2 RPKM values was determined using MS Excel 3.7.

### 2.7. *K*_a_/*K*_s_ Analysis among Domesticated and Wild Eggplants

In genetic analysis, the *K*_a_/*K*_s_ ratio represents the ratio of the number of nonsynonymous substitutions per nonsynonymous site (*K*_a_) to the number of synonymous substitutions per synonymous site (*K*_s_), which could be used as an indicator of selective pressure acting on protein-coding genes. In order to estimate the *K*_a_/*K*_s_ ratio, the bidirectional best hit (BBH) method [27] was used to search the orthologous genes among 6 eggplants. *K*_a_/*K*_s_ calculation was performed with the PAML package using default settings [28]. Comparisons of homologous genes with a high *K*_a_/*K*_s_ ratio significantly >1 are usually considered to be evolving under positive selection, whereas *K*_a_/*K*_s_ratio < 1 indicates purifying selection on the gene loci, and *K*_a_/*K*_s_ ratio close to 1 indicates neutral mutation.

## 3. Results and Discussion

### 3.1. De Novo Transcriptome Sequencing and Assembly of Different Eggplants

Molecular analyses have been conducted to evaluate genetic diversity among wild and domesticated eggplant species using various approaches such as RAPD, SSR, and sequence analysis [29–32]. However, the changes in transcriptional networks and sequence divergence that accompany with domestication remain unknown. To assess selection pressure and the extent of transcriptome variation that occurred during *S. melongena* domestication, the leaf transcriptomes of three *S. melongena* cultivars, HZHQ (MEL_HZHQ), LYQ (MEL_LYQ), and S58 (MEL_S58), and distant relatives, *S. aethiopicum* (AET_SG), *S. integrifolium* (INT_2006), and *S. sisymbriifolium* (SIS_2007), were analyzed.

RNA samples from leaf tissues of each species were prepared for Illumina sequencing to generate the transcriptome data of eggplant. A total of more than 426 million reads were generated (ranging from 55 to 72 million reads for each sample), with 381 million Illumina reads (89.45%) passing the quality filtering. The average Phred quality score was at least 95% for 99% base accuracy for filtered reads, indicating the high quality of filtered reads (Table 1). The Trinity software (trinityrnaseq_r2012-10-05) was used for *de novo* assembly of high-quality clean read sequences into transcript reads, ranging from 73,161 transcripts for *S. sisymbriifolium* to 101,387 for *S. melongena* S58. Only the longest transcripts were defined as final unigenes, yielding 44,073 (*S. sisymbriifolium*) to 58,677 (*S. melongena* S58) unigenes of at least 200 bp in length for all species, with an average length of 408-850 bp and a total length of 36.6-46 Mb (Table 2). N50 being a weighted median statistic such that 50% of the entire assembly is contained in transcripts equal to or larger than this value in bp; The N50 value of the unigenes was between 1,368 bp and 1,606 bp (Table 2). The decrease in N50 values between transcripts and unigenes suggests that longer genes tend to generate more isoforms [33]. Overall, the average number of unigenes for the three *S. melongena* accessions was 55,500. Across the six selected eggplants, the N50 values were between 1,368 bp and 1,576 bp, which are higher than those in Yang et al.'s study [33].

### 3.2. CDS Prediction, Functional Annotation, and Sequence Comparison

Genome annotation was then performed to provide important and pertinent information on gene function and structure. The coding sequence (CDS) of all unigenes was predicted by searching against the NCBI nonredundant (NR) and Swiss-Prot protein databases using ESTScan (3.0.3) or BLAST. The upstream and downstream sequences of the CDS in each unigene were considered as the 5′ and 3′ UTR sequences. 22%-31% unigenes contained the 5′ or 3′ UTR sequence, and 77%-83% CDS sequence among all sample was found from unigenes (Table 3). S58 contained the most unigenes among all the species analyzed, whereas *S. sisymbriifolium* had the highest ratio of UTR and CDS per unigene (Table 3). We annotated between 56.32% (MEL-LYQ) and 66.3% (SIS-2007) unigenes using an *E*-value threshold of 1*e* − 5. The number of annotated genes for *S. Melongena* is comparable to the study published by Yang et al. [33], indicating high quality of the assembly presented in this study. The lack of annotation for some transcripts is due to the number of small transcripts generated in *de novo* assembly that did not show significant similarity with known proteins in various databases.

**Figure 2:**
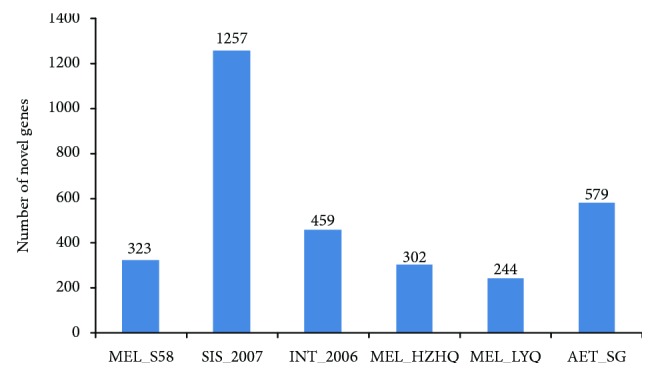
Number of novel transcripts. Histogram of the genes that are not mapped to the current release version of Nakate-Shinkuro reference genome in each of the analyzed samples.

Clean reads for all samples were aligned to the eggplant reference genome (*S. melongena* var. Nakate-Shinkuro) using the TopHat software [13, 34]. The three *S. melongena* cultivars mapped ~88% of the published eggplant genome reads, whereas *S. aethiopicum* and *S. sisymbriifolium* mapped 71% and 27% respectively (Table 3). The mapping results also identified novel transcripts for the six eggplants that are not present in the current release of the eggplant reference genome (Figure 2). As expected, *S. sisymbriifolium* contained the most count of the novel transcripts (1257 genes), followed by *S. aethiopicum* (579 genes), *S. integrifolium* (459 genes), and *S. melongena* cultivars (323 genes for S58, 302 genes for HZHQ, and 244 genes for LYQ).

### 3.3. Characterization of Sequence Diversity among Different Eggplants

**Figure 3:**
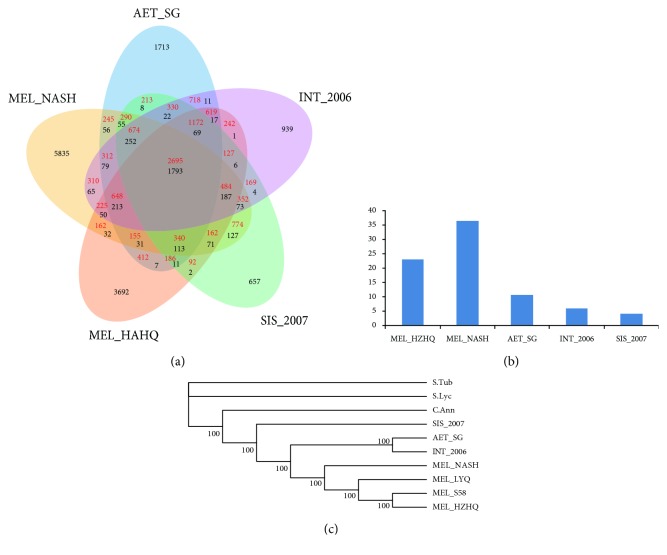
(a) Venn diagram exhibiting the comparison of orthologous gene sequences among the wild *Solanum* species *S. sisymbriifolium* (SIS_2007), *S. aethiopicum* (AET_SG), *S. integrifolium* (INT_2006), and *S. melongena* cultivar HZHQ (MEL_HZHQ) and Nakate-Shinkuro (MEL_NASH) transcriptome. The number of common or specific genes is list in the circle. The shared genes among five species are categorized as single copy (1 : 1, red letters) and multicopy (N : N; black letters). (b) The histogram showing the ratio of species-specific genes. (c) The phylogenetic tree constructed using OrthoMCL based on the sequences of orthologous genes in the six eggplant samples and other Solanaceae family species (*C. annuum*, *S. lycopersicum*, and *S. tuberosum*). All of the nodes have 100% bootstrap support values.

The genus *Solanum* contains up to 1400 species, the two most well-studied crop species tomato and potato are both from the New World, whereas the eggplant is of Old World origin and belongs to the subgenus *Leptostemonum* [12, 33]. The Old World subgenus *Leptostemonum* includes *S. melongena* (section Melongena), *S. macrocarpon L.* (section Melongena), and *S. aethiopicum L.* (section Oliganthes). The origin of the eggplant lineage is most likely started in Africa; however, the relationships among African species and their Asian relatives are not well understood [12]. To further investigate the phylogenetic relationship between species within the Old World clade and that from a more distant clade, we characterized the amount and nature of the genes that are either common or specific to *S. melongena* (HZHQ), *S. aethiopicum*, *S. integrifolium*, and *S. sisymbriifolium*. The published reference eggplant genome (*S. melongena* Nakate-Shinkuro) was also included in the analysis. Orthologous genes among the six species were determined using predicted protein sequences and the OrthoMCL algorithm (OrthoMCL Software-v2.0.3) [16, 35]. Genes from different species were considered as orthologues if the shared homology in their deduced amino acid sequences (BlastP, *e* < 0.00001) was more than 80% of the size of the genes being compared. Overall, 2,695 single-copy genes and 1,793 multicopy genes were common to all five species analyzed (Figure 3(a)). Interestingly, the two *S. melongena* cultivars, Nakate-Shinkuro and HZHQ, had the highest number of specific genes (5,835 and 3,692 respectively, Figure 3(a)) with a ratio of specific to total genes being around 30% (Figure 3(b)). On the other hand, *S. aethiopicum* and *S. sisymbriifolium* had a lower number of species-specific genes with 1,713 and 657 genes, respectively (Figure 3(a)); the number of specific genes for *S. integrifolium* was 939 genes. The results indicate that *S. melongena*, which has been subjected to intense domestication, has seen its pool of new gene sequences increased compared to other relatives. Moreover, the results also support the fact that *S. aethiopicum* has not been subjected to such an intense domestication.

Based on sequence similarity with the genes of other species, the specific genes for the five eggplant species presented in Figure 3(a) were assigned a Gene Ontology (GO) function. Several GO items were not found in wild species especially *S. sisymbriifolium*, such as locomotion in biological process and items within a cellular component, which contain more specific genes and specific GO items (Supplementary Tables S1 and S2). Compared to the distant wild relatives *S. aethiopicum* and *S. sisymbriifolium*, cultivated eggplants have a number of genes with novel GO items such as locomotion, positive regulation of biological process, cell junction, extracellular matrix part, receptor activity, and synapse, which were not found in *S. sisymbriifolium* (Supplementary Table S1). However, the two GO items cell killing and channel regulator activity were lost in cultivar species HZHQ. The data shows that during domestication, eggplant has undergone the evolvement of a more complex molecular and metabolic function. Furthermore, the cultivated eggplant species has experienced rapid and strong adaptation in response to the fluctuating environmental conditions, but at the same time, some functions were lost.

### 3.4. Phylogenetic Relationships among the Wild and Cultivated Eggplants

Bayesian inference methods were then used to construct a phylogenetic tree using the single-copy orthologous gene sequences with the reference genomes of eggplant (Nakate-Shinkuro, MEL_NASH) [13], potato (S*olanum tuberosum*) [36], tomato (*Solanum lycopersicum*) [37], and pepper (*Capsicum annuum*) [10]. The resulting phylogenetic tree (Figure 3(c)) shows that the two domesticated and semiwild cultivars were belonging to the same clade; MEL_HZHQ and S58 were in a sister clade. Two of the wild species, *S. aethiopicum* and *S. integrifolium*, are in a sister clade; this is somehow supporting the classification that *S. integrifolium* is one of the four groups in *S. aethiopicum*, whereas the wild species *S. sisymbriifolium* was most distant from the domesticated species. This category result is consistent with previous reports [12, 19]. Moreover, the cultivated pepper was found more closely related to eggplant than cultivated potato and tomato (Figure 3(c)). However, Wang et al. [38] and Song et al. [18] showed that eggplant was most closely related to potato and tomato clade than pepper. This difference in topologies is possibly resulting from incomplete gene sorting in eggplant leaf transcriptome.

### 3.5. The Expression Divergence among Different Eggplants

**Figure 4:**
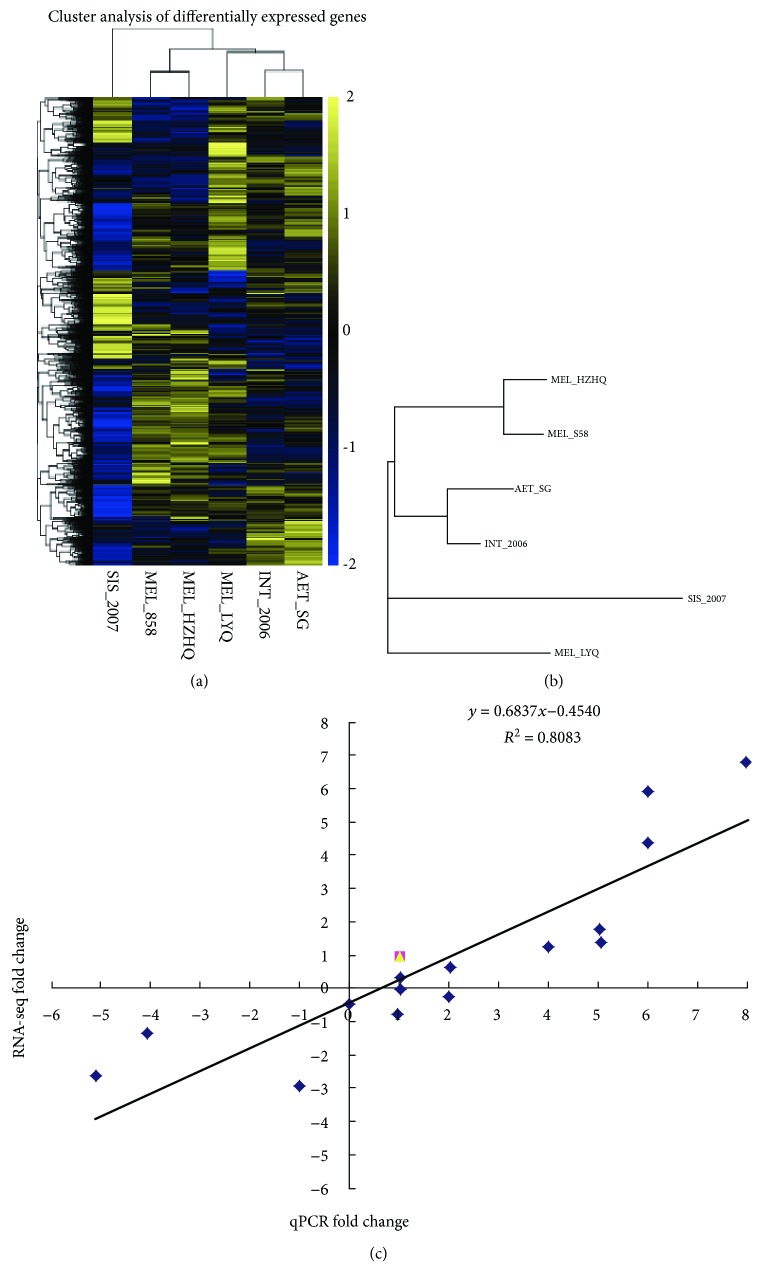
(a) The heatmap depicting expression profiles of orthologous genes in the six eggplant species *S. sisymbriifolium* (SIS_2007), *S. aethiopicum* (AET_SG), *S. integrifolium* (INT_2006), and *S. melongena* cultivars HZHQ (MEL_HZHQ), LYQ (MEL_LYQ), and S58 (MEL_S58). Scaled log2 expression values are shown with yellow and blue indicating high and low expressions, respectively. (b) Hierarchical clustering of the differentially expressed genes using the RNA-seq data derived from six samples based on RPKM values. (c) Correlation of fold change values from RNA-seq and qPCR based on the SmActin expression. The *R* ^2^ value is 0.808.

We further examined the differential expression in leaf tissues of the six eggplant accessions and detected the expression of orthologous genes. Interspecific comparisons based on pairwise gene expression differences revealed a striking decrease in the *S. sisymbriifolium* (SIS_2007) gene expression branch length (Figure 4(a)). Hierarchical clustering of the expression profiles in leaf tissues of all species showed that the two cultivated eggplants HZHQ and S58 were included into the same sister cluster, whereas two wild relatives *S. aethiopicum* (AET_SG) and *S. integrifolium* (INT_2006) were in the same cluster (Figure 4(b)). The results revealed that species within the same clade has similar gene expression pattern.

We compared gene expression data to identify genes showing evidence of differential expression among different eggplants. Wild relatives of cultivated crops usually possess allele resistance to pathogens, diseases, and extreme environment stresses. For example, *S. aethiopicum* provides source of abiotic and biotic resistance for cultivated *S. melongena*, and *S. sisymbriifolium* is the source of bioactive compounds [39–43]. In the present study, the number of genes with expression changes and KEGG pathway was significantly increased in all comparisons with the wild *S. sisymbriifolium* than any other lineages (Table 4). KEGG pathway enrichment analysis of these genes revealed overrepresentation of identified genes involved in stress response, metabolism, defense response, photosynthesis, response to pathogen, and redox pathways (Supplementary Table S3). Moreover, we identified genes involved in plant-pathogen interaction and plant hormone signal transduction pathway in the upregulation comparisons between other eggplants and *S. sisymbriifolium* (highlighted in red; Supplementary Table S3). Therefore, we propose that the transcriptional scope of *S. sisymbriifolium* is highly diverged from other five eggplants and that environmental stress has played a major role in driving transcriptional variations among different eggplants.

To validate the RNA-seq data, qPCR of 16 randomly selected genes was performed. As shown in Figure 4(c), there was a strong positive correlation (*R* ^2^ = 0.81) between RNA-seq data and qPCR data. The qPCR expression quantities were basically consistent with their transcript abundance changes identified by RNA-seq, which confirms the reliability of RNA-seq data.

### 3.6. Analysis of Positive Selection in the Wild and Cultivated Eggplant

The genetic basis of domestication-related traits has been studied in several organisms, including important crops such as maize, millet, rice, and cucumber [4, 44–46]. It is proposed that the rapid phenotypic divergence associated with domestication is often controlled by a limited number of genetic loci [6]. In maize, population genetic analysis and whole-genome resequencing have revealed that a small proportion of genes/genome region (~5%) show evidence of positive selection during domestication [44, 47]. To assess selection pressure and the extent of transcriptome variation which occurred during *S. melongena* domestication, the leaf transcriptome of the six eggplant accessions was analyzed. The ratio of *K*_a_/*K*_s_ by random substitution throughout the coding gene within specific loci can be used to estimate selective pressure; a gene with *K*_a_/*K*_s_ > 1 indicates that this gene is subjected to positive selection, whereas *K*_a_/*K*_s_ < 1 indicates purifying selection, and *K*_a_/*K*_s_ score close to 1 indicates neutral mutation [48]. From comparison of gene-level estimates of *K*_a_/*K*_s_ in all species, we identified 19 unigenes (listed in Table 5) that exhibited statistically significant (*P* < 0.05) evidence of evolution under positive selection across the phylogeny. Many of these genes have been annotated in tomato and potato and characterized in details in rice and *Arabidopsis*. The result supports the hypothesis that relatively few mutations have gone through strong selective sweeps during domestication.

The 19 genes subjected to positive selection could be divided into four groups: hormone response, development and response to disease and abiotic stress, oxidation-reduction pathway, and development. Orthologous genes *OG06215*, *OG08277*, and *OG12205* are likely related to the hormone response. *OG06215* is characterized as a Domon superfamily which is auxin-induced protein in root cultures. *OG08277* is a ribosomal protein, with annotated functions of controlling vacuole trafficking and developmental programs through the regulation of lipid metabolism in *Arabidopsis*. Ribosome proteins serve as translational regulators of auxin response, and lipid metabolism modulates auxin-mediated tissue differentiation [49]. Auxin plays a major role in the dynamic and complex phytohormone regulatory networks controlling fruit development [50]. In the present study, *OG12205* was identified as an Aux/IAA family gene; its orthologous genes in tomato interact with ARF (auxin response factor) proteins and involved in the regulation of quality parameters over tomato fruit development and play diverse roles in flower and fruit development [51–53].

Many gene/protein families are reported to be associated with stress tolerance and disease resistance, such as PPR (pentatricopeptide repeat) protein, *WRKY*, E3 ubiquitin ligase, and DnaJ homolog subfamily proteins [54–59]. Park et al. [60] found that cleavage of PPR protein mRNAs renders *Arabidopsis thaliana* more susceptible to pathogenic bacteria and fungi. PPR proteins also involved in chloroplast, mitochondrial and seed development, and abiotic stress response in rice and maize [5, 53, 54, 61]. DnaJ homolog subfamily proteins as cochaperones also have critical functions in biotic and abiotic stress response. Overexpression of tomato chloroplast-targeted DnaJ protein enhances tolerance to drought stress and resistance to *Pseudomonas solanacearum* in transgenic tobacco [62]. We identified eight genes with annotated functions of response to disease and abiotic stress in other organisms (Table 5). For example, the gene *OG0372* functions as pentatricopeptide repeat-containing protein; one gene (*OG10870*) belongs to the DnaJ homolog subfamily. A series of positive genes were annotated as hydrothermal carbonisation (HTC) in leaves or roots and involved in the oxidation-reduction pathway, such as *OG06179*, *OG06849*, and *OG05048*. *OG09458* belongs to ubiquinone oxidoreductase, also functions in HTC in the leaf. *OG05094* is annotated as 2-aminoethanethiol dioxygenase-like protein. In addition, there were three development-related genes found to have been subjected to positive selection, a thiamin pyrophosphokinase (TPK) encoding gene (*OG07167*) and two Ca2+-dependent phospholipid-binding protein encoding genes (*OG06647* and *OG05019*).

*OG07232* and *OG07554* were E3 ubiquitin ligases which respond to drought stress in maize [59] and involved in rice antiviral defense in the early stages of rice dwarf virus infection [63]. We identified two F-box/kelch repeat proteins *OG09141* and *OG08464* that are positively selected. *OG06038* and *OG03732* were annotated as Calvin cycle protein and early nodulin 16 precursor, respectively, which also involved in plant response to stresses and rapid environmental changes [64–67]. However, further experiments and field observations are needed to discuss whether/how the functions of those positively selected genes were promoted during domestication.

## 4. Conclusions

Previous studies have shown that domestication-related traits are often controlled by few genetic loci. However, the selective sweeps in eggplants remain unknown. We identified 44,073-58,677 unigenes for six wild and cultivated eggplants; the average number of unigenes for the three *S. melongena* species (55,500 genes) and the N50 values (1,368~1,576 bp) are both higher than that for the recent transcriptomic study on eggplant [33]. The phylogenetic tree revealed that the three cultivated eggplants in section Melongena (HZHQ, LYQ, and S58) were grouped into the same clade; *S. aethiopicum* and *S. integrifolium* from section Oliganthes were included in the sister clade, close to the cultivated eggplants, whereas the wild species *S. sisymbriifolium* in the Sisymbriifolium clade was in the most distant clade from the domesticated species. Functional analysis of species-specific genes indicate that eggplant has gone through complex molecular and metabolic changes during domestication, evolving its own gene pool with increased new genes as well as rapid adaptation to different environmental conditions. Moreover, relatively few mutations (19 positively selected genes) have gone through strong selective sweeps during eggplant domestication. Our results provide insights into the selection patterns of the transcriptomes of wild and cultivated eggplants and the understanding of complex domesticated-related traits.

## Figures and Tables

**Table 1:** Information of RNA-seq data for the six eggplants.

Sample	Raw reads	Clean reads	GC (%)	Phred quality Q20 (%)		Total mapped^a^
				-1	-2	
AET_SG	61795572	55580924	41.99	97.32	95.85	39337841 (70.78%)
INT_2006	74782564	67387058	42.51	97.21	96.03	48949822 (72.64%)
MEL_HZHQ	65869048	58960600	41.68	97.28	96.09	51659443 (87.62%)
MEL_LYQ	66197610	59365428	42.35	97.27	95.81	52647559 (88.68%)
MEL_S58	75562708	67428684	41.68	97.3	95.93	58745699 (87.12%)
SIS_2007	82147678	72679508	42.45	97.18	96.10	20027221 (27.56%)
Total	426355180	381402202				

^a^The number of high-quality reads mapped using TopHat and the ratio with the clean reads.

**Table 2:** Summary feature of the eggplant transcriptome assemblies.

Sample	Transcript				Unigene			
	Number	Total length (Mb)	Average length (bp)	N50 (bp)	Number	Total length (Mb)	Average length (bp)	N50 (bp)
AET_SG	100296	13.3	1322	2186	51519	43.8	850	1576
INT_2006	82045	10.1	1239	2025	45838	40.5	463	1606
MEL_HZHQ	88368	98.4	1113	1876	55118	43.1	420	1368
MEL_LYQ	98897	12.4	1257	2081	52819	44.8	430	1568
MEL_S58	101387	12.1	1193	2010	58677	46	408	1408
SIS_2007	73161	77.1	1053	1716	44073	36.6	450	1469

**Table 3:** The number and ratio of the CDS, annotated genes, and mapped sequences for the six eggplants.

Sample	5′ UTR^A^	3′ UTR^A^	CDS		Total CDS ratio(%)^B^	Number of unigenes annotated^C^	Total mapped^D^
			Blast	EST Scan			
AET_SG	12123 (23.53%)	11626 (22.57%)	21195	18652	77.34	29118 (56.51%)	39337841 (70.78%)
INT_2006	12830 (27.99%)	12393 (27.04%)	20952	15564	79.66	28321 (61.78%)	48949822 (72.64%)
MEL_HZHQ	14230 (25.82%)	13570 (24.62%)	26867	18952	83.13	34865 (63.25%)	51659443 (87.62%)
MEL_LYQ	12908 (24.44%)	12405 (23.49%)	21749	19162	77.45	29751 (56.32%)	52647559 (88.68%)
MEL_S58	13867 (23.63%)	12992 (22.14%)	26486	20652	80.33	35659 (60.77%)	58745699 (87.12%)
SIS_2007	13523 (30.68%)	14051 (31.88%)	24478	11525	81.69	29221 (66.3%)	20027221 (27.56%)

^A^The number of 5′ UTR or 3′ UTR and the ratio with the unigenes. ^B^The total CDS ratio with the unigenes. ^C^The number of unigenes annotated to the database and the ratio with the all unigenes. ^D^The number of high-quality reads mapped to the reference eggplant genome and the ratio with the clean reads.

**Table 4:** The number of genes with expression changes in interspecific comparisons.

Comparison sample name	DEG^a^ number
MEL_HZHQ-AET_SG	3578
MEL_HZHQ-INT_2006	2955
MEL_HZHQ-SIS_2007	5834
MEL_LYQ-AET_SG	3007
MEL_LYQ-INT_2006	3110
MEL_LYQ-SIS_2007	6473
MEL_S58-INT_2006	2734
MEL_S58-SIS_2007	5678
SIS_2007-AET_SG	5591
SIS_2007-INT_2006	4302

^a^DEG: differentially expressed genes.

**Table 5:** Genes subjected to positive selection in the domestication of eggplant.

Function classification	Orthologous gene ID	*K*_a_/*K*_s_	Gene name or protein family	Function annotated	References
Hormone response	OG06215	1.27994	Domon superfamily	Auxin-induced protein in root culture protein	
	OG12205	1.45957	Aux/IAA family	Auxin-responsive protein	Kumar et al. [51]
	OG08277	1.77181	Ribosomal protein	Serve as translational regulators of auxin response	Li et al. [54]

Response to disease and abiotic stress	OG03724	1.30858	Pentatricopeptide repeat protein	Respond to disease and abiotic stress, such as pathogenic bacteria, fungi, and ABA. Play a broad and essential role in plant growth and development	Park et al. [60], Tan et al. [55], Li et al. [49]
	OG07232, OG07554	1.74664	E3 ubiquitin ligase	Respond to drought stress and antiviral defense. Involve in the postgerminative seedling growth transition and root and flowering development.	Xia et al. [59], Liu et al. [63]
		1.02972			
	OG10870	1.55109	DnaJ subfamily	Enhances tolerance to drought stress and Pseudomonas solanacearum	Wang et al. [62]
	OG09141	1.13547	F-box/kelch repeat protein	Promotes nematode susceptibility and involved in light signaling, flowering, and circadian control. Regulates leaf senescence, seed size, and panicle architecture and modulates cytokinin levels	Curtis et al. [68]
	OG08464	1.59571			Chen et al. [69]
	OG06038	1.4737	Calvin cycle protein CP12 superfamily	Linked to stress responses and be part of a redox-mediated metabolic switch, allowing organisms to respond to rapid changes in the external environment	Gontero and Maberly [65], Marri et al. [66], López et al. [67]
	OG03732	1.22007	Early nodulin 16 precursor	Involved in various plant activities and plant response to environmental factors such as light, water deficit, cold, ozone stress and toxicity, and osmotic stress	Wu et al. [64]

Oxidation reduction pathway	OG06179	1.15523	HTC in leaf	Nitrogen compound metabolic process and oxidation-reduction process and nitrite reductase (cytochrome, ammonia-forming) activity	
	OG06849	1.23173	HTC in root, Clp protease proteolytic subunit 3	Thioredoxin-like protein and cell redox homeostasis	
	OG05048	1.03583		Thioredoxin serine dehydrogenase proteinase	
				Proteolysis serine-type endopeptidase activity	
	OG09458	1.65912	HTC in leaf, NADH: ubiquinone oxidoreductase	Transport proton when NADH was dehydrogenated to ubiquinone in mitochondria	
	OG05094	1.04564	Aminoethanethiol dioxygenase	Cysteine dioxygenase activity and L-cysteine oxidation-reduction	

Development	OG05091	1.12163	Thiamin pyrophosphokinase	TPK activity for thiamin cofactor activation and this activity is essential for viability	Ajjawi et al. [70]
	OG06647, OG05019	5.73809	Phospholipid-binding protein	Critical for maintaining directional root hair growth in Arabidopsis	Yoo et al. [71]
		1.04878			

The references in which the homolog of the eggplant unigene in other species had been characterized for its biological function.

## Data Availability

The raw data of RNA-seq experiment is deposited in the Sequence Read Archive (NCBI): SRP127743.

## Supplementary Materials

Supplementary Materials Table S1: detailed GO annotation of species-specific genes for the three wild eggplant species and two cultivars. Table S2: specific GO terms for the three wild eggplant species and two cultivars. Table S3: overrepresented KEGG pathway enrichment among genes with higher expression based on the interspecific pairwise gene expression comparisons. Table S4: primer sequences for the 16 random selected genes used in qPCR analysis.
